# In vivo and in vitro performance of a China-made hemodialysis machine: a multi-center prospective controlled study

**DOI:** 10.1186/s12938-017-0387-y

**Published:** 2017-08-02

**Authors:** Yong Wang, Xiang-Mei Chen, Guang-Yan Cai, Wen-Ge Li, Ai-Hua Zhang, Li-Rong Hao, Ming Shi, Rong Wang, Hong-Li Jiang, Hui-Min Luo, Dong Zhang, Xue-Feng Sun

**Affiliations:** 1 Department of Nephrology, Chinese PLA General Hospital, Chinese PLA Institute of Nephrology, State Key Laboratory of Nephrology, National Clinical Research Center for Chronic Kidney Diseases, No. 28 Fuxing Road, Haidian District, Beijing, 100853 China; 2 Department of Nephrology, China-Japan Friendship Hospital, Beijing, 100029 China; 3 Department of Nephrology, Peking University Third Hospital, Beijing, 100191 China; 4 Department of Nephrology, the First Affiliated Hospital of Harbin Medical University, Harbin, 150001 China; 5 Department of Nephrology, Renmin Hospital of Wuhan University, Wuhan, 430060 China; 6 Department of Nephrology, Shandong Provincial Hospital, Jinan, 250021 China; 7 Department of Nephrology, the First Affiliated Hospital of Xi’an Medical University, Xi’an, 710061 China; 8 Department of Nephrology, First People’s Hospital of Yunnan Province, Kunming, 650032 China

**Keywords:** Hemodialysis machine, Performance, Safety, China-made

## Abstract

**Objective:**

To evaluate the in vivo and in vitro performance of a China-made dialysis machine (SWS-4000).

**Methods:**

This was a multi-center prospective controlled study consisting of both long-term in vitro evaluations and cross-over in vivo tests in 132 patients. The China-made SWS-4000 dialysis machine was compared with a German-made dialysis machine (Fresenius 4008) with regard to Kt/V values, URR values, and dialysis-related adverse reactions in patients on maintenance hemodialysis, as well as the ultrafiltration rate, the concentration of electrolytes in the proportioned dialysate, the rate of heparin injection, the flow rate of the blood pump, and the rate of malfunction.

**Results:**

The Kt/V and URR values at the 1st and 4th weeks of dialysis as well as the incidence of adverse effects did not differ between the two groups in cross-over in vivo tests (*P* > 0.05). There were no significant differences between the two groups in the error values of the ultrafiltration rate, the rate of heparin injection or the concentrations of electrolytes in the proportioned dialysate at different time points under different parameter settings. At weeks 2 and 24, with the flow rate of the blood pump set at 300 mL/min, the actual error of the SWS-4000 dialysis machine was significantly higher than that of the Fresenius 4008 dialysis machine (*P* < 0.05), but there was no significant difference at other time points or under other settings (*P* > 0.05). The malfunction rate was higher in the SWS-4000 group than in the Fresenius 4008 group (*P* < 0.05).

**Conclusions:**

The in vivo performance of the SWS-4000 dialysis machine is roughly comparable to that of the Fresenius 4008 dialysis machine; however, the malfunction rate of the former is higher than that of the latter in in vitro tests. The stability and long-term accuracy of the SWS-4000 dialysis machine remain to be improved.

## Background

The incidence of end-stage renal disease (ESRD) has risen over the past several decades globally [1]. Hemodialysis has become the standard treatment for ESRD and saved many patients’ life. Statistics from the Chinese National Renal Data System (CNRDS) show that more than 300,000 uremic patients are currently receiving hemodialysis treatment and the number of patients in need of hemodialysis may be as high as one to two million in the near future [2]. However, most patients in China cannot afford regular hemodialysis treatment due to its high cost. An important reason for the high cost of hemodialysis is that hemodialysis equipment and related products are largely imported. Since hemodialysis equipment accounts for the largest proportion of expense of hemodialysis treatment, domestication of hemodialysis equipment is a key step to lower the cost of hemodialysis.

Hemodialysis machine is an instrument integrating various technologies including computer, electronics, mechanics, fluid dynamics, biochemistry, optics and acoustics, among others. Due to technological reasons, foreign brands have long dominated in China’s hemodialysis equipment market [2]. After years of development, some China’s companies have succeeded in breaking the monopoly of foreign companies and developed several brands of domestic hemodialysis machines with independent intellectual property rights. However, the smaller size and sales, and fewer service and maintenance sites of these domestic manufacturers make their hemodialysis products less competitive on the market [3–5]. More importantly, the clinical performance of these domestic hemodialysis systems varies greatly and lacks rigorous evaluation, which greatly hampers their marketing and clinical application. Although there have been few studies comparing the clinical performance of China-made and imported hemodialysis machines in recent years, they are only single-center clinical studies conducted in patients [6, 7]. There is still an urgent need to comprehensively evaluate the clinical performance and safety of China-made hemodialysis machines.

Fresenius 4008 and SWS-4000 dialysis machines are, respectively, the most commonly used imported and domestic brands in China. According to statistics from the CNRDS, Fresenius 4008 and SWS-4000 dialysis machines have a market share of 45.4 and 1.9%, respectively, in China [2, 8]. By referring to the China’s national standard “Requirements for the Safety of Hemodialysis, Hemodiafiltration and Hemofiltration Device” [9, 10], we performed this large-scale multi-center prospective controlled study to evaluate the in vivo and in vitro performance, stability, and durability of SWS-4000 dialysis machine, with an aim to get a better understanding of the quality of domestic hemodialysis machines to facilitate their wide use and help find what should be improved for them.

## Methods

### Study design

This was a multi-center prospective controlled study conducted in eight centers, with both in vivo and in vitro studies performed to compare the in vivo and in vitro performance of SWS-4000 dialysis machines made by Chongqing Shanwaishan Technology Co., Ltd (Chongqing, China) and Fresenius 4008 dialysis machines made by a German company (Fresenius, Germany). The study was approved by the respective institutional ethics committee of each participating center. Informed consent was obtained from each participant in the in vivo study.

### In vivo study

Patients undergoing maintenance hemodialysis at the participating hospitals were enrolled in this study. The inclusion criteria were: (1) Age ≥18 years old; (2) hemodialysis duration >3 months; (3) maintenance hemodialysis three times per week; and (4) written informed consent. The exclusion criteria were: (1) acute renal failure; (2) planned kidney transplantation within 1 year; (3) switch to peritoneal dialysis; (4) positivity for hepatitis B virus, hepatitis C virus or human immunodeficiency virus; (5) severe diseases such as cancer, severe infection, cirrhosis, and congestive heart failure; (6) poor compliance and failure to follow the study protocol; and (7) pregnancy or breastfeeding. The exit criteria were: (1) being unable to continue hemodialysis; (2) violation of the requirements for treatment; (3) request by patients, investigators or sponsors; and (4) termination of the study by the institutional ethics committee.

Patients meeting the inclusion and exclusion criteria were randomly divided into either an SWS-4000 group or a Fresenius 4008 group according to the hemodialysis system used. After the initial treatment for 6 weeks, the two groups were switched and continued the treatment for another 6 weeks. Patients in the same dialysis center underwent dialysis using the same dialysis machines and dialysate, and only SWS-4000 or Fresenius 4008 dialysis machine was allowed to use. When bicarbonate dialysate was used, dry dialysate was not allowed to use, and the flow rate was set at 500 mL/h, with other therapeutic regimens unchanged.

At the 1st and 4th weeks of dialysis, blood Na^+^, K^+^, Ca^2+^, HCO^3−^, BUN and Cr levels were determined to calculate Kt/V and URR according to the reported formulae [11]. The changes in blood Na^+^, K^+^, Ca^2+^, HCO^3−^, BUN and Cr levels relative to pre-dialysis values were also evaluated. Dialysis-related adverse reactions were recorded to assess the safety of the dialysis machines.

### In vitro study

For the in vitro study, two domestic SWS-4000 dialysis machines and two imported Fresenius 4008 dialysis machines were tested at each of the dialysis centers. The performance of dialysis machines was evaluated on non-therapeutic days for 24 weeks. Outcome measures included accuracy of in vitro hemodialysis ultrafiltration (dehydration), the concentration of electrolytes in dialysate, accuracy of the heparin pump, accuracy of the flow rate of the blood pump, endotoxin detection, and bacterial culture.

The accuracy of in vitro hemodialysis ultrafiltration (dehydration) was assessed using reverse osmosis water on Sundays of the 1st, 12th, and 23rd weeks. In the ISO-UF mode with the ultrafiltration rate set at 200, 400, 600 and 1000 mL/h, the machines were run for one hour, respectively. The collected filtrate was measured with a measuring cup to calculate the ultrafiltration rate, and the error value was calculated as the difference between the measurement value and the set value.

In the second dialysis in the 1st, 4th, 8th, 12th, 16th, 20th, and 24th weeks, the concentration of electrolytes in the proportioned dialysate was detected, after which dialysate specimens were collected for endotoxin detection and routine bacterial culture. The error value was calculated as the difference between the measurement value and the nominal value.

The accuracy of the heparin pump was assessed using reverse osmosis water on the same days as the assessment of the ultrafiltration performance. Setting the heparin injection rate set at 2 and 4 mL/h, the machines were run for 2 h, respectively. The collected reverse osmosis water was measured with a measuring cup to calculate the heparin injection rate, and the error value was calculated as the difference between the measurement value and the set value.

The accuracy of the flow rate of the blood pump was assessed using reverse osmosis water on Sundays of the 2nd, 13th, and 24th weeks. Setting the flow rate set at 100, 200, 300, and 400 mL/min, the machines were run for 1 h, respectively. The collected reverse osmosis water was measured with a measuring cup to calculate the flow rate, and the error value was calculated as the difference between the measurement value and the set value.

In addition, malfunction and maintenance were recorded throughout the 24-week study period. The malfunction rate is calculated as the number of times of malfunctions divided by the total number of rounds of dialysis performed.

### Statistical analysis

Statistical analyses were performed using SPSS 13.0 software. Numerical data are expressed as the mean ± standard deviation (SD) if they followed a normal distribution; otherwise they are expressed as median with minimum (min) and maximum (max). Paired *t* test, analysis of variance (ANOVA) and *q* test were used to analyze inter-group differences for data with homogeneous variance, while nonparametric rank test was used for data without homogeneous variance. Count data are expressed as percentages and were compared using χ^2^ test (or Fisher exact test). *P* values <0.05 were considered statistically significant.

## Results

### Demographic characteristics of the study population

This clinical trial was carried out at eight dialysis centers. We planned to enroll 160 patients, but only 134 were initially enrolled. Two participants dropped out of the clinical trial. Thus, a total of 132 participants completed the clinical trial, of whom 80 were male and 52 were female. They ranged in age from 21 to 83 years, with a mean age of 52.8 ± 15.3 years. In terms of primary disease, there were 58 cases of chronic glomerulonephritis, 25 cases of diabetic nephropathy, 6 cases of polycystic kidney disease, 20 cases of hypertensive renal damage, and 23 cases of other kidney diseases.

### In vivo study

**Fig. 1:**
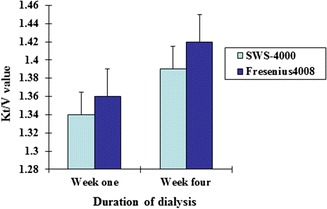
Comparison of changes in Kt/V values after dialysis between the China-made and imported dialysis machines

**Fig. 2:**
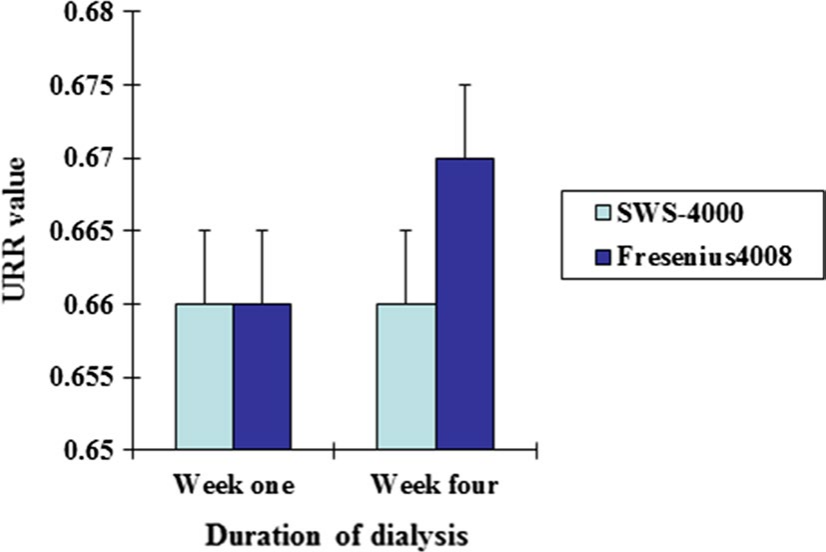
Comparison of changes in URR values after dialysis between the China-made and imported dialysis machines

**Fig. 3:**
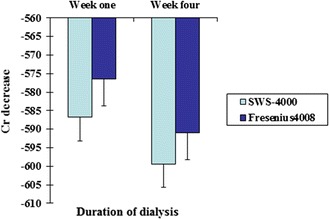
Comparison of blood Cr changes after dialysis between the China-made and imported dialysis machines

**Fig. 4:**
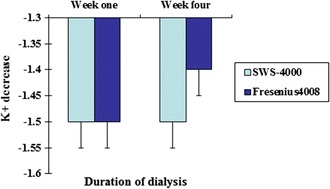
Comparison of blood K^+^ changes after dialysis between the China-made and imported dialysis machines

As shown in Table 1 and Figs. 1, 2, there was no significant difference in Kt/V or URR values at the 1st and 4th weeks of dialysis between the two groups (*P* > 0.05). There was also no significant difference in the changes in blood creatinine, K^+^ or HCO^3−^ level at 1 and 4 weeks after dialysis between the two groups (*P* > 0.05; Table 2; Figs. 3, 4).

**Table 1:** Comparison of changes in Kt/V and URR values after dialysis between the two types of dialysis machines

	SWS-4000		Fresenius 4008	
	Week 1	Week 4	Week 1	Week 4
Kt/V	1.34 ± 0.49	1.39 ± 0.66	1.36 ± 0.44	1.42 ± 0.64
URR	0.66 ± 0.08	0.66 ± 0.95	0.66 ± 0.08	0.67 ± 0.09

**Table 2:** Comparison of changes in blood biochemical parameters after dialysis between the two types of dialysis machines

	SWS-4000		Fresenius 4008	
	Week 1	Week 4	Week 1	Week 4
Cr decline	586.8 ± 168.8	599.4 ± 183.8	576.4 ± 171.3	590.9 ± 174.9
K^+^ decline	1.5 ± 0.6	1.5 ± 0.6	1.5 ± 0.5	1.4 ± 0.6
HCO_3_^−^ change	4.7 ± 3.5	4.9 ± 3.5	5.3 ± 3.8	6.3 ± 7.3

A total of 2382 rounds of dialysis were performed on the domestic SWS-4000 dialysis machine, during which seven adverse events occurred, including three cases of hypotension, two cases of hypertension, and two cases of muscle spasm. As a comparison, three adverse events developed during a total of 2376 rounds of dialysis performed on the imported Fresenius 4008 dialysis machine, including two cases of hypotension and one case of hypertension. There was no significant difference in the rate of adverse events between the two groups (*P* > 0.05).

### In vitro study

Following the testing protocol, a total of 16 SWS-4000 dialysis machines and 16 Fresenius 4008 dialysis machines were tested in the in vitro study. To assess the performance of the ultrafiltration pump, the error values of the ultrafiltration rate were determined. At all time points and the set ultrafiltration rates, the ultrafiltration error values for all dialysis machines were lower than 30 mL/h. As shown in Table 3, there was no significant difference in the ultrafiltration error values between the SWS-4000 dialysis machine and Fresenius 4008 dialysis machine at different ultrafiltration rates (*P* > 0.05).

**Table 3:** Ultrafiltration errors for the two types of dialysis machines at different time points under different ultrafiltration rate settings

Test time	Ultrafiltration rate (mL/h)	Actual error (ml)	
		SWS-4000	Fresenius 4008
Week 1	200	7.9 ± 12.0	6.6 ± 8.6
	400	10.5 ± 17.9	4.4 ± 6.4
	600	4.9 ± 5.4	6.4 ± 10.0
	1000	20.5 ± 23.2	14.1 ± 3.8
Week 12	200	8.2 ± 11.8	6.8 ± 9.6
	400	8.4 ± 12.5	4.9 ± 9.7
	600	16.6 ± 27.5	8.8 ± 12.5
	1000	14.1 ± 15.4	14.6 ± 14.0
Week 23	200	10.7 ± 14.5	10.6 ± 13.7
	400	16.0 ± 20.0	6.6 ± 13.2
	600	11.3 ± 13.2	8.8 ± 13.7
	1000	16.8 ± 16.3	25.1 ± 27.9

In order to assess the performance of the proportioning pump, the primary dialysate was allowed to pass through the proportioning pump and then the concentrations of different ions in the proportioned dialysis solution were measured. As shown in Table 4, there was no significant difference in the concentrations of various ions at different time points in the dialysis solutions that have passed through the two dialysis machines (*P* > 0.05).

**Table 4:** Errors of ion concentrations in proportioned dialysate for the two types of dialysis machines at different time points

Test time	Ion (mmol/L)	Actual error (ml)	
		SWS-4000	Fresenius 4008
Week 1	K^+^	0.12 ± 0.10	0.09 ± 0.10
	Na^+^	1.40 ± 1.74	1.45 ± 1.37
	Cl^−^	1.40 ± 1.40	1.35 ± 0.94
	Ga^2+^	0.06 ± 0.05	0.04 ± 0.05
	HCO_3_^−^	1.69 ± 1.73	1.45 ± 0.91
Week 4	K^+^	0.11 ± 0.10	0.11 ± 0.11
	Na^+^	1.89 ± 1.80	2.65 ± 3.07
	Cl^−^	2.06 ± 2.20	2.43 ± 2.04
	Ga^2+^	0.06 ± 0.05	0.05 ± 0.05
	HCO_3_^−^	1.54 ± 1.45	1.96 ± 1.82
Week 8	K^+^	0.09 ± 0.10	0.13 ± 0.11
	Na^+^	1.37 ± 1.53	1.95 ± 1.18
	Cl^−^	1.55 ± 1.97	1.84 ± 0.87
	Ga^2+^	0.11 ± 0.11	0.05 ± 0.05
	HCO_3_^−^	2.06 ± 1.41	1.29 ± 1.25
Week 12	K^+^	0.10 ± 0.11	0.10 ± 0.10
	Na^+^	1.93 ± 1.43	1.70 ± 1.63
	Cl^−^	2.05 ± 1.70	1.23 ± 1.03
	Ga^2+^	0.05 ± 0.05	0.03 ± 0.03
	HCO_3_^−^	1.70 ± 1.92	1.14 ± 0.89
Week 16	K^+^	0.10 ± 0.11	0.13 ± 0.11
	Na^+^	3.08 ± 1.74	2.26 ± 1.73
	Cl^−^	1.98 ± 1.91	1.60 ± 1.18
	Ga^2+^	0.08 ± 0.09	0.06 ± 0.06
	HCO_3_^−^	1.24 ± 0.90	1.15 ± 1.58
Week 20	K^+^	0.17 ± 0.15	0.15 ± 0.16
	Na^+^	2.04 ± 1.33	1.88 ± 1.51
	Cl^−^	3.05 ± 2.66	1.66 ± 1.77
	Ga^2+^	0.07 ± 0.06	0.03 ± 0.04
	HCO_3_^−^	2.36 ± 1.85	2.04 ± 2.69
Week 24	K^+^	0.13 ± 0.10	0.16 ± 0.24
	Na^+^	3.20 ± 2.64	2.03 ± 2.42
	Cl^−^	1.93 ± 2.22	2.25 ± 1.91
	Ga^2+^	0.10 ± 0.07	0.06 ± 0.05
	HCO_3_^−^	1.61 ± 1.78	2.52 ± 2.48

The performance of the heparin pump and blood pump was also assessed. As shown in Table 5, at different time points under different heparin injection speed settings, the error values of the heparin injection rate for all dialysis machines were less than 0.2 mL/h, and there was no significant difference between the two types of dialysis machines (*P* > 0.05). Table 6 shows the error values of the flow rate of the blood pump for all dialysis machines at different time points under different flow rate settings. The error values of the flow rate of the blood pump for all dialysis machines were less than 10 mL/min. At weeks 2 and 24 with the flow rate set at 300 mL/min, the actual error of the SWS-4000 dialysis machine was higher than that of the Fresenius 4008 dialysis machine (*P* < 0.05), but there was no significant difference between the actual errors of the blood pumps of the two dialysis machines at other time points (*P* > 0.05).

**Table 5:** Errors of heparin injection rate for the two types of dialysis machines at different time points under different injection settings

Test time	Heparin pump (mL/h)	Actual error (ml)	
		SWS-4000	Fresenius 4008
Week 1	2	0.0	0.0
	4	0.04 ± 0.13	0.03 ± 0.08
Week 12	2	0.0	0.0
	4	0.04 ± 0.07	0.01 ± 0.05
Week 23	2	0.10 ± 0.21	0.0
	4	0.13 ± 0.23	0.08 ± 0.19

**Table 6:** Errors of blood pump flow rate for the two types of dialysis machines at different time points under different settings

Test time	Blood pump flow rate (mL/min)	Actual error (ml)	
		SWS-4000	Fresenius 4008
Week 2	100	69.6 ± 128.7	41.8 ± 78.2
	200	146.9 ± 212.3	79.5 ± 76.1
	300	307.5 ± 320.5	86.0 ± 86.5*
	400	385.4 ± 451.8	287.8 ± 334.3
Week 13	100	80.4 ± 118.1	20.6 ± 37.6
	200	175.0 ± 191.3	118.8 ± 155.1
	300	326.3 ± 501.3	133.4 ± 179.2
	400	475.8 ± 517.9	258.2 ± 213.5
Week 24	100	95.7 ± 115.6	65.0 ± 80.7
	200	220.6 ± 253.3	118.1 ± 158.5
	300	449.0 ± 291.7	139.4 ± 121.6*
	400	736.0 ± 701.5	328.1 ± 402.6

* *P* < 0.05, vs. SWS-4000 at the same flow rate

We also assessed potential endotoxin and bacterial contaminations for the two types of dialysis machines. A total of seven rounds of endotoxin and bacterial contamination assessments were performed in seven dialysis centers within 24 weeks. The results showed that no endotoxin or bacterial contaminations occurred in dialysis buffers of both dialysis machines.

### Malfunction rate

A total of 4608 rounds of hemodialysis were performed on 16 SWS-4000 hemodialysis machines within 24 weeks, and 23 times of machine malfunctions were reported, with a malfunction rate of 0.50%. Also, a total of 4608 rounds of hemodialysis were performed on 16 Fresenius 4008 dialysis machines, and 7 times of machine malfunctions were reported, with a malfunction rate of 0.15%. There was a significant difference in the malfunction rates between the two dialysis machine models (*P* < 0.01), indicating that the Fresenius 4008 dialysis machine has fewer times of malfunctions and is more reliable.

## Discussion

In the present study, we performed both long-term in vitro evaluations and cross-over in vivo tests in patients to comprehensively assess the in vitro and in vivo performance of the China-made SWS-4000 hemodialysis machine. We found that this domestic brand had roughly comparable in vivo performance to the German-made Fresenius 4008 hemodialysis system, although the malfunction rate of the former is higher than that of the latter in in vitro tests.

In the in vivo study on patients, we found that there was no significant difference in the efficiency of solute clearance between domestic and imported dialysis machines at weeks 1 and 4 after the initiation of dialysis, i.e., there was no significant difference in major dialysis outcome parameters such as Kt/V and URR [8]. After a single treatment with either machine, BUN and Cr in patients were significantly decreased and the electrolyte and acid–base imbalances were corrected significantly. With the extended treatment cycle, the therapeutic effect of the domestic dialysis machine remained stable and there was no performance degradation. In addition, there was no significant difference in the incidence of complications such as hypotension, hypertension and muscle spasms. These results suggest that patients can achieve good therapeutic effects with both types of hemodialysis machines and that the domestic hemodialysis machine can fully meet the clinical requirements.

**Fig. 5:**
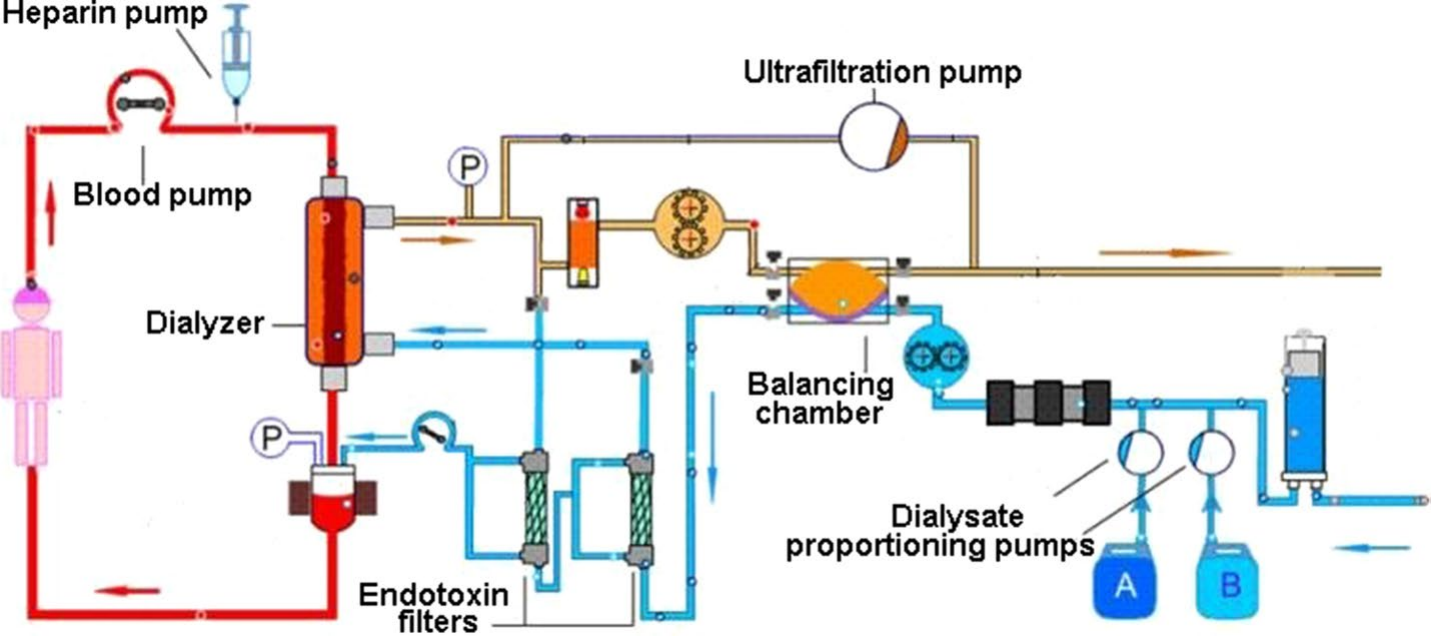
The structural design of SWS-4000 dialysis machine

**Fig. 6:**
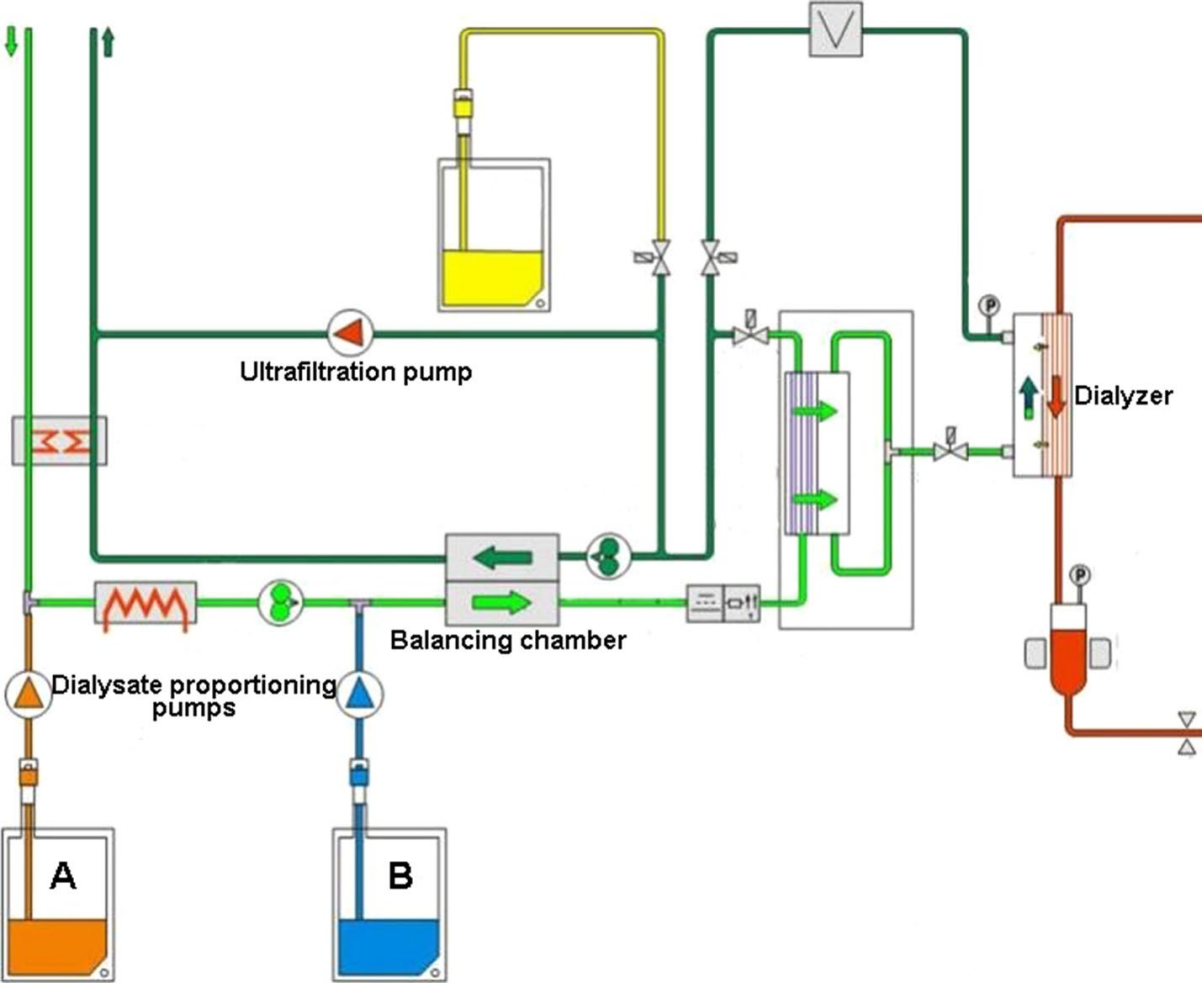
The structural design of Fresenius 4008 dialysis machine

Figures 5 and 6 show the structural design of SWS-4000 and Fresenius 4008 hemodialysis systems, respectively. The core components of a hemodialysis machine that are associated with clinical outcomes include the ultrafiltration system, the dialysate proportioning pump, the blood pump, and the heparin pump, the accuracy and long-term stability of which directly affect patient outcomes [12, 13]. In order to examine if there is any gap in these core components between the domestic and imported hemodialysis machines, we designed 24 weeks of in vitro experiments to evaluate the quality of the domestic hemodialysis machine more objectively.

The accuracy of the ultrafiltration system, which is configured to remove/deliver excess fluid from/to the dialyser, is crucial for patients [14], and it must be ensured that the relative deviation of the measurement value from the set value is not greater than ±1%, or the absolute error is less than ±30 mL/h. The ultrafiltration control system of currently marketed dialysis machines can be divided into two categories: flow sensor system and balancing chamber system. Although both of them have their advantages and disadvantages, ultrafiltration volume is calculated in a same manner for both types of machines. For the Fresenius dialysis machine, fresh dialysis fluid pushes the balancing chamber membrane to expel the waste fluid, and then the same amount of waste fluid pushes the membrane to force fresh dialysis fluid into the balancing chamber. For the SWS-4000 hemodialysis machine, a balanced feedback volume control system is used, and its permitted ultrafiltration error is ±30 mL/h. Our in vitro results showed that the ultrafiltration system of the SWS-4000 hemodialysis machine worked well and showed no difference in long-term evaluations compared with the Fresenius dialysis machine, suggesting that its accuracy and reliability are comparable to those of the Fresenius dialysis machine.

In the functional assessment of a hemodialysis machine, electric conductivity is usually used to assess the function of the dialysate proportioning pump, which accurately mixes precise quantities of water and concentrated dialysate [15, 16]. However, we think that is more clinically meaningful to detect whether the electrolyte concentration in dialysate after proportioning is close to the indicated value. For this purpose, we allowed each dialysis center to use their routinely used dialysate to eliminate the possible influence of different dialysates. Although differences were observed in the dialysate electrolyte concentrations among different dialysis centers, there was no difference in the dialysate electrolyte concentrations between the two dialysis machine models. However, with the extension of the application time, the actual error value of the SWS-4000 hemodialysis machine increased. Although the ceramic metering pump used in the SWS-4000 hemodialysis machine is an imported product, our result suggests that there is still room for improvement of the assembly and overall coordination of the domestic machine to increase their durability. In addition, the results of endotoxin detection and bacterial cultures for both types of machines were all negative, indicating that the safety of the proportioning system in the SWS-4000 domestic dialysis machine is similar to that of the imported dialysis machine and can meet clinical needs.

According to the China’s national standard, the actual flow rate of the blood pump, which controls the extracorporeal circulation of blood, should be within the allowable range from the set value, i.e., a relative error of less than ±10% or an absolute error of less than ±10 m/min. For the SWS-4000 hemodialysis machine in which a self-developed blood pump is equipped, the permissible error is ±10 mL/min. Our test results showed that in the early test stage, the actual error value of the blood pump of the SWS-4000 hemodialysis machine was in line with the national standard, but with extension of the application, the error showed a tendency to increase, indicating that compared with imported dialysis machines, the long-term accuracy of the blood pump of the SWS-4000 hemodialysis machine needs to be improved. The China’s national standard also states that the error for the actual heparin injection rate from the set rate should be less than ±0.2 mL/h or within ±5%. Our results showed that there was no significant difference in the mean errors between the domestic and imported dialysis machines, indicating that the accuracy of the heparin pump of the SWS-4000 hemodialysis machine works well, and this may be because the heparin pump used in the SWS-4000 hemodialysis machine is an imported product.

In this study, we found that the malfunction rate of the SWS-4000 hemodialysis machine was higher, and main malfunctions included false alarms of the blood leaking and air monitoring systems which have been reported previously [17], self-test failure of the balancing system, and multiple replacements of quick coupling and bubble sensor. This result indicates that there are still gaps in software matching and manufacture technology between the domestic and imported dialysis machines, and the long-term reliability of domestic hemodialysis machines remains to be verified.

To the best of our knowledge, this is the first large-scale multi-center study to assess the quality of a China-made hemodialysis machine, in which we took full consideration of the actual operating conditions of each dialysis center. In order not to affect the conventional treatment plan for patients and to maximize the best interests of patients, we could not ensure that the two types of dialysis machines had exactly the same number of work hours when the trial began. In addition, the in vivo test duration was short. Future studies are needed to carefully address these issues and confirm our findings.

## Conclusions

To conclude, there is no significant difference in the in vivo performance or major in vitro parameters between the domestic SWS-400 and the imported Fresenius 4008 hemodialysis machine, and the domestic hemodialysis machine can replace the imported one to meet the basic clinical needs of patients on dialysis. However, the malfunction rate of the domestic hemodialysis machine is still relatively high, and its reliability and long-term accuracy remain to be improved.
